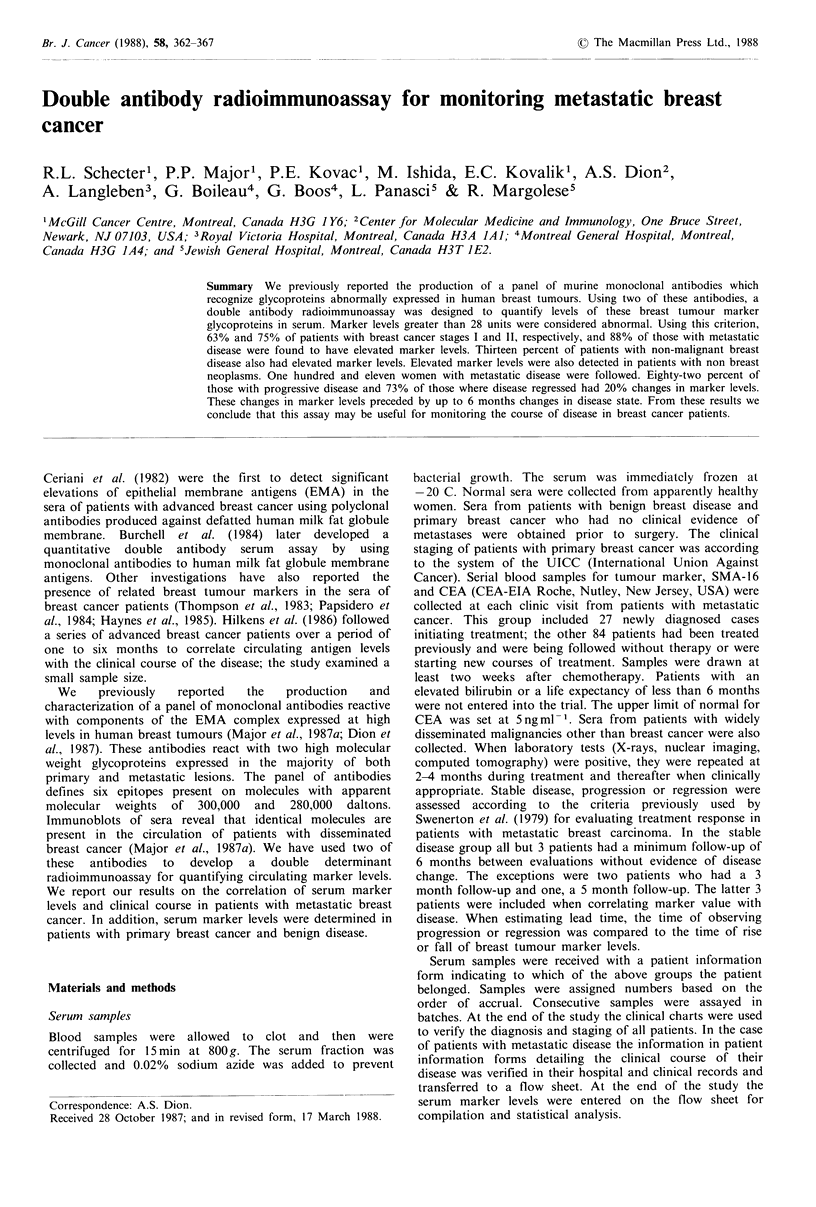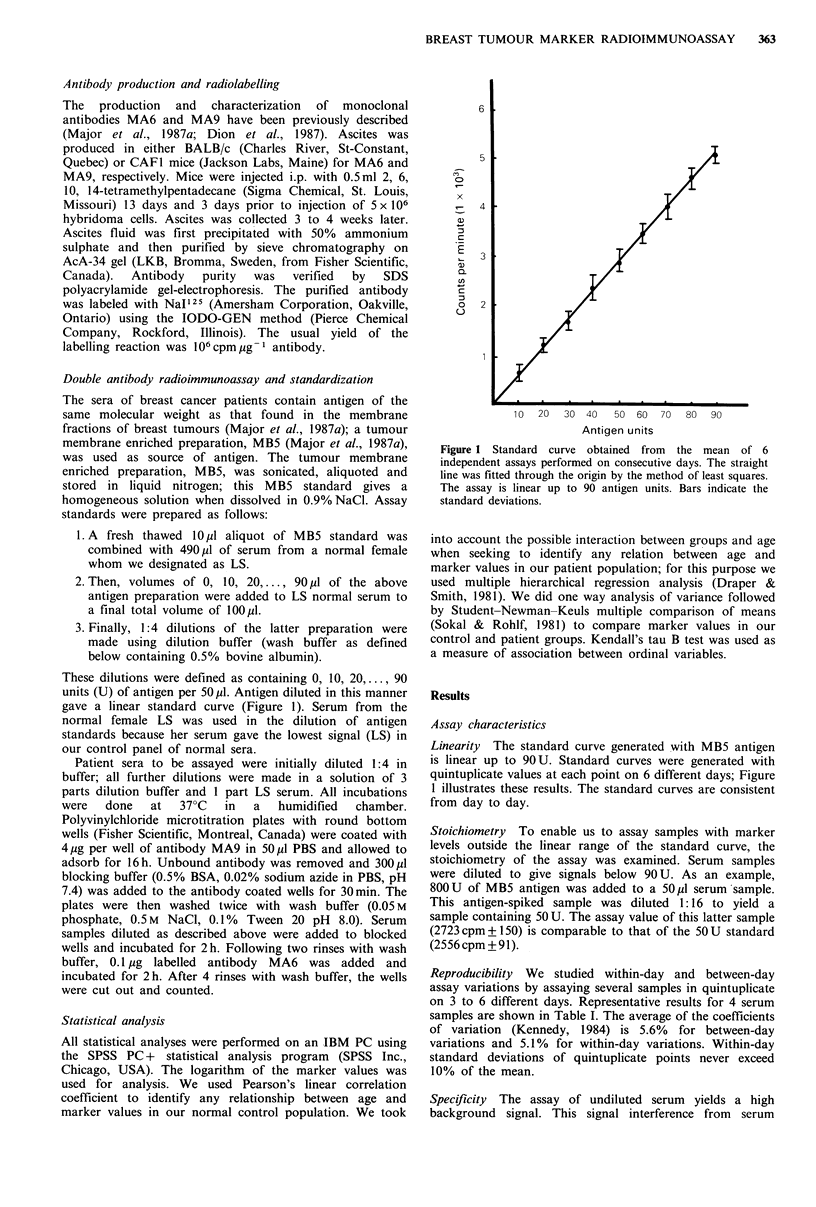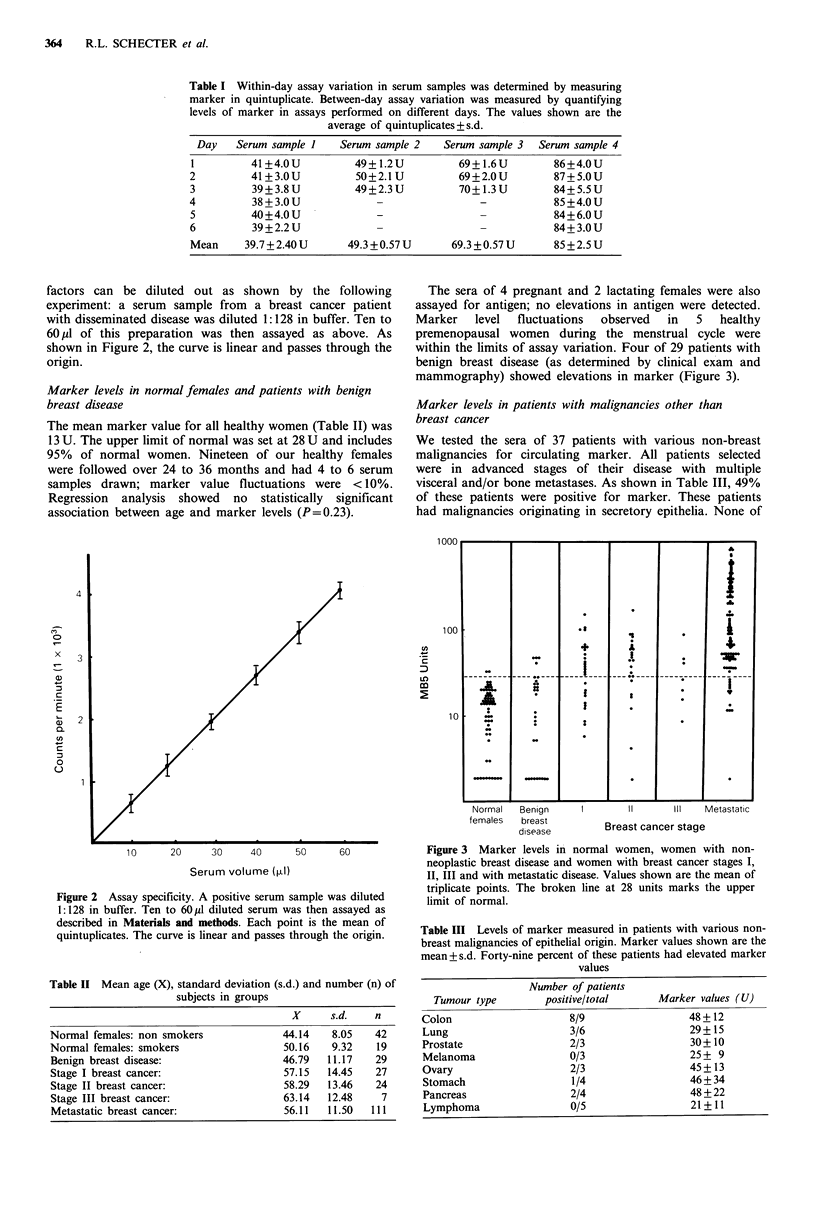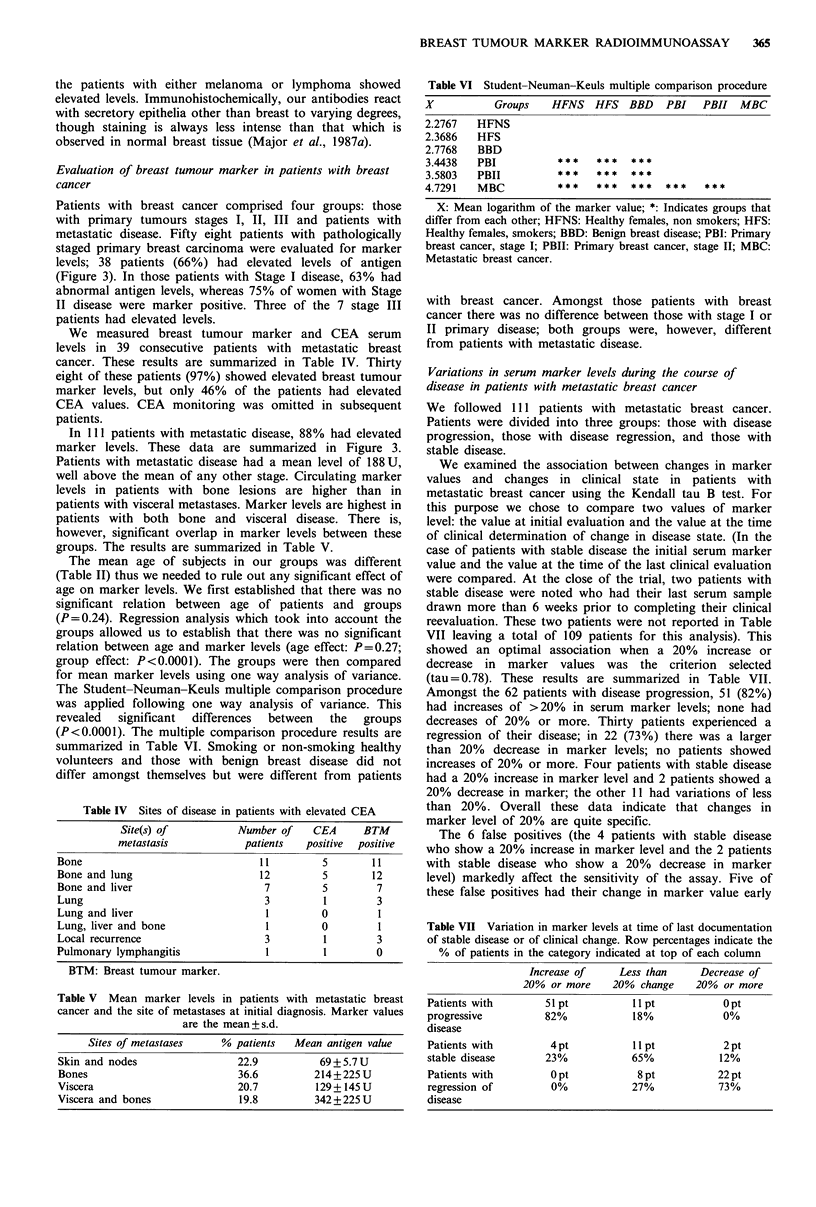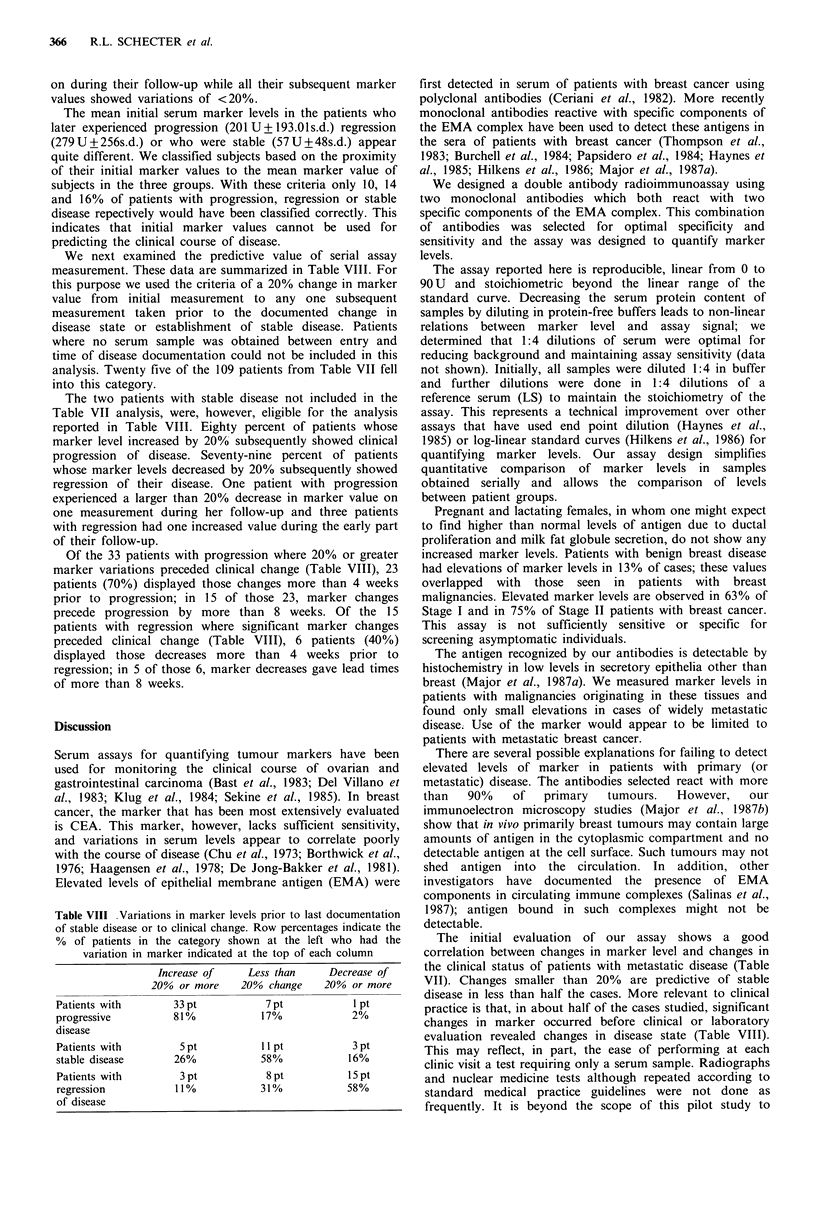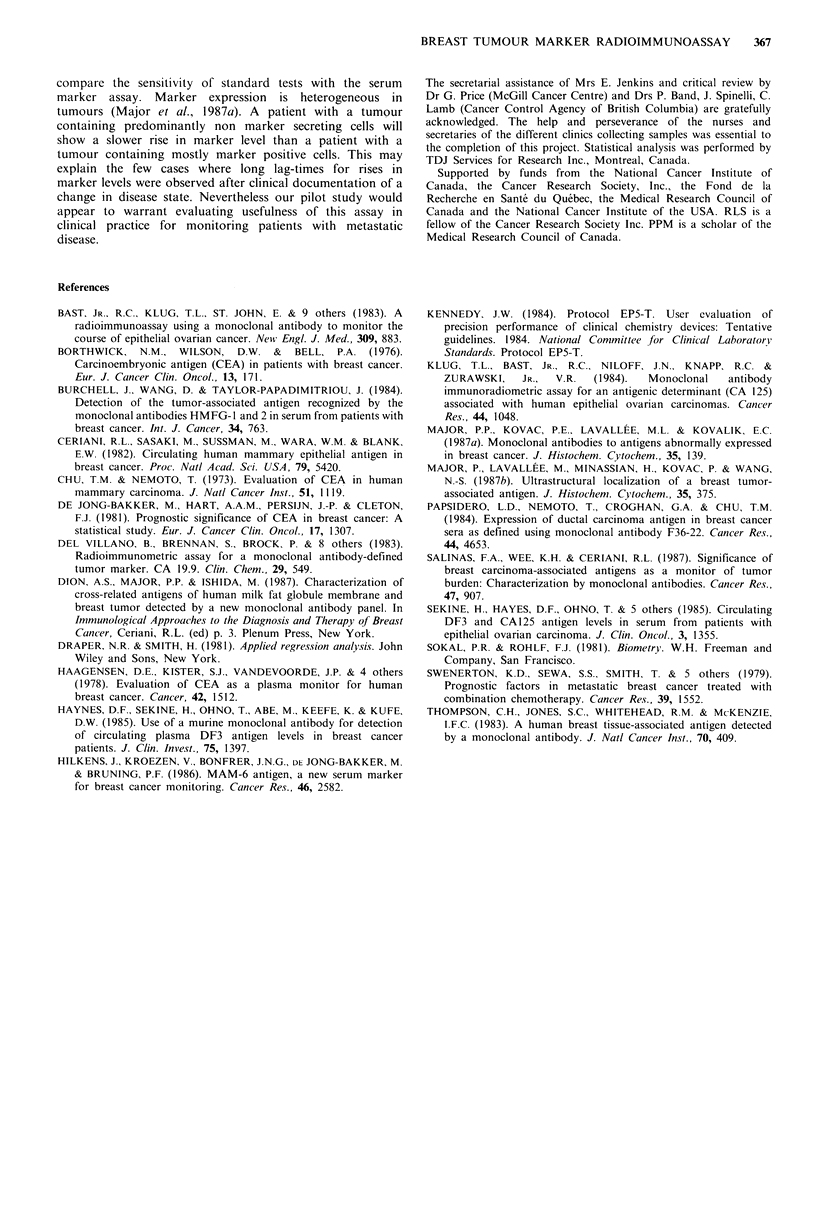# Double antibody radioimmunoassay for monitoring metastatic breast cancer.

**DOI:** 10.1038/bjc.1988.220

**Published:** 1988-09

**Authors:** R. L. Schecter, P. P. Major, P. E. Kovac, M. Ishida, E. C. Kovalik, A. S. Dion, A. Langleben, G. Boileau, G. Boos, L. Panasci

**Affiliations:** McGill Cancer Centre, Montreal, Canada.

## Abstract

We previously reported the production of a panel of murine monoclonal antibodies which recognize glycoproteins abnormally expressed in human breast tumours. Using two of these antibodies, a double antibody radioimmunoassay was designed to quantify levels of these breast tumour marker glycoproteins in serum. Marker levels greater than 28 units were considered abnormal. Using this criterion, 63% and 75% of patients with breast cancer stages I and II, respectively, and 88% of those with metastatic disease were found to have elevated marker levels. Thirteen percent of patients with non-malignant breast disease also had elevated marker levels. Elevated marker levels were also detected in patients with non breast neoplasms. One hundred and eleven women with metastatic disease were followed. Eighty-two percent of those with progressive disease and 73% of those where disease regressed had 20% changes in marker levels. These changes in marker levels preceded by up to 6 months changes in disease state. From these results we conclude that this assay may be useful for monitoring the course of disease in breast cancer patients.


Br. J. Cancer (1988), 58, 362–367 © The Macmillan Press Ltd., 1988

### Double antibody radioimmunoassay for monitoring metastatic breast cancer

R.L. Schecter[1], P.P. Major[1], P.E. Kovac[1], M. Ishida, E.C. Kovalik[1], A.S. Dion[2], A. Langleben[3], G. Boileau[4], G. Boos[4], L. Panasci[5] & R. Margolese[5]

[1]*McGill Cancer Centre, Montreal, Canada H3G 1Y6;* [2]*Center for Molecular Medicine and Immunology, One Bruce Street, Newark, NJ 07103, USA;* [3]*Royal Victoria Hospital, Montreal, Canada H3A 1A1;* [4]*Montreal General Hospital, Montreal, Canada H3G 1A4; and* [5]*Jewish General Hospital, Montreal, Canada H3T 1E2.*

**Summary** We previously reported the production of a panel of murine monoclonal antibodies which recognize glycoproteins abnormally expressed in human breast tumours. Using two of these antibodies, a double antibody radioimmunoassay was designed to quantify levels of these breast tumour marker glycoproteins in serum. Marker levels greater than 28 units were considered abnormal. Using this criterion, 63% and 75% of patients with breast cancer stages I and II, respectively, and 88% of those with metastatic disease were found to have elevated marker levels. Thirteen percent of patients with non-malignant breast disease also had elevated marker levels. Elevated marker levels were also detected in patients with non breast neoplasms. One hundred and eleven women with metastatic disease were followed. Eighty-two percent of those with progressive disease and 73% of those where disease regressed had 20% changes in marker levels. These changes in marker levels preceded by up to 6 months changes in disease state. From these results we conclude that this assay may be useful for monitoring the course of disease in breast cancer patients.

Ceriani *et al.* (1982) were the first to detect significant elevations of epithelial membrane antigens (EMA) in the sera of patients with advanced breast cancer using polyclonal antibodies produced against defatted human milk fat globule membrane. Burchell *et al.* (1984) later developed a quantitative double antibody serum assay by using monoclonal antibodies to human milk fat globule membrane antigens. Other investigations have also reported the presence of related breast tumour markers in the sera of breast cancer patients (Thompson *et al.*, 1983; Papsidero *et al.*, 1984; Haynes *et al.*, 1985). Hilkens *et al.* (1986) followed a series of advanced breast cancer patients over a period of one to six months to correlate circulating antigen levels with the clinical course of the disease; the study examined a small sample size.

We previously reported the production and characterization of a panel of monoclonal antibodies reactive with components of the EMA complex expressed at high levels in human breast tumours (Major *et al.*, 1987*a*; Dion *et al.*, 1987). These antibodies react with two high molecular weight glycoproteins expressed in the majority of both primary and metastatic lesions. The panel of antibodies defines six epitopes present on molecules with apparent molecular weights of 300,000 and 280,000 daltons. Immunoblots of sera reveal that identical molecules are present in the circulation of patients with disseminated breast cancer (Major *et al.*, 1987*a*). We have used two of these antibodies to develop a double determinant radioimmunoassay for quantifying circulating marker levels. We report our results on the correlation of serum marker levels and clinical course in patients with metastatic breast cancer. In addition, serum marker levels were determined in patients with primary breast cancer and benign disease.

### Materials and methods

### *Serum samples*

Blood samples were allowed to clot and then were centrifuged for 15 min at 800*g*. The serum fraction was collected and 0.02% sodium azide was added to prevent bacterial growth. The serum was immediately frozen at −20 C. Normal sera were collected from apparently healthy women. Sera from patients with benign breast disease and primary breast cancer who had no clinical evidence of metastases were obtained prior to surgery. The clinical staging of patients with primary breast cancer was according to the system of the UICC (International Union Against Cancer). Serial blood samples for tumour marker, SMA-16 and CEA (CEA-EIA Roche, Nutley, New Jersey, USA) were collected at each clinic visit from patients with metastatic cancer. This group included 27 newly diagnosed cases initiating treatment; the other 84 patients had been treated previously and were being followed without therapy or were starting new courses of treatment. Samples were drawn at least two weeks after chemotherapy. Patients with an elevated bilirubin or a life expectancy of less than 6 months were not entered into the trial. The upper limit of normal for CEA was set at $5\,ng\,ml^{-1}$. Sera from patients with widely disseminated malignancies other than breast cancer were also collected. When laboratory tests (X-rays, nuclear imaging, computed tomography) were positive, they were repeated at 2–4 months during treatment and thereafter when clinically appropriate. Stable disease, progression or regression were assessed according to the criteria previously used by Swenerton *et al.* (1979) for evaluating treatment response in patients with metastatic breast carcinoma. In the stable disease group all but 3 patients had a minimum follow-up of 6 months between evaluations without evidence of disease change. The exceptions were two patients who had a 3 month follow-up and one, a 5 month follow-up. The latter 3 patients were included when correlating marker value with disease. When estimating lead time, the time of observing progression or regression was compared to the time of rise or fall of breast tumour marker levels.

Serum samples were received with a patient information form indicating to which of the above groups the patient belonged. Samples were assigned numbers based on the order of accrual. Consecutive samples were assayed in batches. At the end of the study the clinical charts were used to verify the diagnosis and staging of all patients. In the case of patients with metastatic disease the information in patient information forms detailing the clinical course of their disease was verified in their hospital and clinical records and transferred to a flow sheet. At the end of the study the serum marker levels were entered on the flow sheet for compilation and statistical analysis.

Correspondence: A.S. Dion.
Received 28 October 1987; and in revised form, 17 March 1988.

*Antibody production and radiolabelling*

The production and characterization of monoclonal antibodies MA6 and MA9 have been previously described (Major *et al.*, 1987*a*; Dion *et al.*, 1987). Ascites was produced in either BALB/c (Charles River, St-Constant, Quebec) or CAF1 mice (Jackson Labs, Maine) for MA6 and MA9, respectively. Mice were injected i.p. with 0.5 ml 2, 6, 10, 14-tetramethylpentadecane (Sigma Chemical, St. Louis, Missouri) 13 days and 3 days prior to injection of $5 \times 10^6$ hybridoma cells. Ascites was collected 3 to 4 weeks later. Ascites fluid was first precipitated with 50% ammonium sulphate and then purified by sieve chromatography on AcA-34 gel (LKB, Bromma, Sweden, from Fisher Scientific, Canada). Antibody purity was verified by SDS polyacrylamide gel-electrophoresis. The purified antibody was labeled with $NaI^{125}$ (Amersham Corporation, Oakville, Ontario) using the IODO-GEN method (Pierce Chemical Company, Rockford, Illinois). The usual yield of the labelling reaction was $10^6$ cpm $\mu g^{-1}$ antibody.

*Double antibody radioimmunoassay and standardization*

The sera of breast cancer patients contain antigen of the same molecular weight as that found in the membrane fractions of breast tumours (Major *et al.*, 1987*a*); a tumour membrane enriched preparation, MB5 (Major *et al.*, 1987*a*), was used as source of antigen. The tumour membrane enriched preparation, MB5, was sonicated, aliquoted and stored in liquid nitrogen; this MB5 standard gives a homogeneous solution when dissolved in 0.9% NaCl. Assay standards were prepared as follows:

1. A fresh thawed 10 μl aliquot of MB5 standard was combined with 490 μl of serum from a normal female whom we designated as LS.
2. Then, volumes of 0, 10, 20,..., 90 μl of the above antigen preparation were added to LS normal serum to a final total volume of 100 μl.
3. Finally, 1:4 dilutions of the latter preparation were made using dilution buffer (wash buffer as defined below containing 0.5% bovine albumin).

These dilutions were defined as containing 0, 10, 20,..., 90 units (U) of antigen per 50 μl. Antigen diluted in this manner gave a linear standard curve (Figure 1). Serum from the normal female LS was used in the dilution of antigen standards because her serum gave the lowest signal (LS) in our control panel of normal sera.

Patient sera to be assayed were initially diluted 1:4 in buffer; all further dilutions were made in a solution of 3 parts dilution buffer and 1 part LS serum. All incubations were done at 37°C in a humidified chamber. Polyvinylchloride microtitration plates with round bottom wells (Fisher Scientific, Montreal, Canada) were coated with 4 μg per well of antibody MA9 in 50 μl PBS and allowed to adsorb for 16 h. Unbound antibody was removed and 300 μl blocking buffer (0.5% BSA, 0.02% sodium azide in PBS, pH 7.4) was added to the antibody coated wells for 30 min. The plates were then washed twice with wash buffer (0.05 M phosphate, 0.5 M NaCl, 0.1% Tween 20 pH 8.0). Serum samples diluted as described above were added to blocked wells and incubated for 2 h. Following two rinses with wash buffer, 0.1 μg labelled antibody MA6 was added and incubated for 2 h. After 4 rinses with wash buffer, the wells were cut out and counted.

*Statistical analysis*

All statistical analyses were performed on an IBM PC using the SPSS PC+ statistical analysis program (SPSS Inc., Chicago, USA). The logarithm of the marker values was used for analysis. We used Pearson's linear correlation coefficient to identify any relationship between age and marker values in our normal control population. We took into account the possible interaction between groups and age when seeking to identify any relation between age and marker values in our patient population; for this purpose we used multiple hierarchical regression analysis (Draper & Smith, 1981). We did one way analysis of variance followed by Student–Newman–Keuls multiple comparison of means (Sokal & Rohlf, 1981) to compare marker values in our control and patient groups. Kendall's tau B test was used as a measure of association between ordinal variables.

**Figure 1** Standard curve obtained from the mean of 6 independent assays performed on consecutive days. The straight line was fitted through the origin by the method of least squares. The assay is linear up to 90 antigen units. Bars indicate the standard deviations.

### Results

*Assay characteristics*

*Linearity* The standard curve generated with MB5 antigen is linear up to 90 U. Standard curves were generated with quintuplicate values at each point on 6 different days; Figure 1 illustrates these results. The standard curves are consistent from day to day.

*Stoichiometry* To enable us to assay samples with marker levels outside the linear range of the standard curve, the stoichiometry of the assay was examined. Serum samples were diluted to give signals below 90 U. As an example, 800 U of MB5 antigen was added to a 50 μl serum sample. This antigen-spiked sample was diluted 1:16 to yield a sample containing 50 U. The assay value of this latter sample (2723 cpm ± 150) is comparable to that of the 50 U standard (2556 cpm ± 91).

*Reproducibility* We studied within-day and between-day assay variations by assaying several samples in quintuplicate on 3 to 6 different days. Representative results for 4 serum samples are shown in Table I. The average of the coefficients of variation (Kennedy, 1984) is 5.6% for between-day variations and 5.1% for within-day variations. Within-day standard deviations of quintuplicate points never exceed 10% of the mean.

*Specificity* The assay of undiluted serum yields a high background signal. This signal interference from serum

**Table I** Within-day assay variation in serum samples was determined by measuring marker in quintuplicate. Between-day assay variation was measured by quantifying levels of marker in assays performed on different days. The values shown are the average of quintuplicates ± s.d.

| Day | Serum sample 1 | Serum sample 2 | Serum sample 3 | Serum sample 4 |
|---|---|---|---|---|
| 1 | 41±4.0 U | 49±1.2 U | 69±1.6 U | 86±4.0 U |
| 2 | 41±3.0 U | 50±2.1 U | 69±2.0 U | 87±5.0 U |
| 3 | 39±3.8 U | 49±2.3 U | 70±1.3 U | 84±5.5 U |
| 4 | 38±3.0 U | – | – | 85±4.0 U |
| 5 | 40±4.0 U | – | – | 84±6.0 U |
| 6 | 39±2.2 U | – | – | 84±3.0 U |
| Mean | 39.7±2.40 U | 49.3±0.57 U | 69.3±0.57 U | 85±2.5 U |

factors can be diluted out as shown by the following experiment: a serum sample from a breast cancer patient with disseminated disease was diluted 1:128 in buffer. Ten to 60 μl of this preparation was then assayed as above. As shown in Figure 2, the curve is linear and passes through the origin.

*Marker levels in normal females and patients with benign breast disease*

The mean marker value for all healthy women (Table II) was 13 U. The upper limit of normal was set at 28 U and includes 95% of normal women. Nineteen of our healthy females were followed over 24 to 36 months and had 4 to 6 serum samples drawn; marker value fluctuations were <10%. Regression analysis showed no statistically significant association between age and marker levels ($P = 0.23$).

**Figure 2** Assay specificity. A positive serum sample was diluted 1:128 in buffer. Ten to 60 μl diluted serum was then assayed as described in **Materials and methods**. Each point is the mean of quintuplicates. The curve is linear and passes through the origin.

**Table II** Mean age (X), standard deviation (s.d.) and number (n) of subjects in groups

| | X | s.d. | n |
|---|---|---|---|
| Normal females: non smokers | 44.14 | 8.05 | 42 |
| Normal females: smokers | 50.16 | 9.32 | 19 |
| Benign breast disease: | 46.79 | 11.17 | 29 |
| Stage I breast cancer: | 57.15 | 14.45 | 27 |
| Stage II breast cancer: | 58.29 | 13.46 | 24 |
| Stage III breast cancer: | 63.14 | 12.48 | 7 |
| Metastatic breast cancer: | 56.11 | 11.50 | 111 |

The sera of 4 pregnant and 2 lactating females were also assayed for antigen; no elevations in antigen were detected. Marker level fluctuations observed in 5 healthy premenopausal women during the menstrual cycle were within the limits of assay variation. Four of 29 patients with benign breast disease (as determined by clinical exam and mammography) showed elevations in marker (Figure 3).

*Marker levels in patients with malignancies other than breast cancer*

We tested the sera of 37 patients with various non-breast malignancies for circulating marker. All patients selected were in advanced stages of their disease with multiple visceral and/or bone metastases. As shown in Table III, 49% of these patients were positive for marker. These patients had malignancies originating in secretory epithelia. None of

**Figure 3** Marker levels in normal women, women with non-neoplastic breast disease and women with breast cancer stages I, II, III and with metastatic disease. Values shown are the mean of triplicate points. The broken line at 28 units marks the upper limit of normal.

**Table III** Levels of marker measured in patients with various non-breast malignancies of epithelial origin. Marker values shown are the mean ± s.d. Forty-nine percent of these patients had elevated marker values

| Tumour type | Number of patients positive/total | Marker values (U) |
|---|---|---|
| Colon | 8/9 | 48±12 |
| Lung | 3/6 | 29±15 |
| Prostate | 2/3 | 30±10 |
| Melanoma | 0/3 | 25± 9 |
| Ovary | 2/3 | 45±13 |
| Stomach | 1/4 | 46±34 |
| Pancreas | 2/4 | 48±22 |
| Lymphoma | 0/5 | 21±11 |

the patients with either melanoma or lymphoma showed elevated levels. Immunohistochemically, our antibodies react with secretory epithelia other than breast to varying degrees, though staining is always less intense than that which is observed in normal breast tissue (Major *et al.*, 1987*a*).

*Evaluation of breast tumour marker in patients with breast cancer*

Patients with breast cancer comprised four groups: those with primary tumours stages I, II, III and patients with metastatic disease. Fifty eight patients with pathologically staged primary breast carcinoma were evaluated for marker levels; 38 patients (66%) had elevated levels of antigen (Figure 3). In those patients with Stage I disease, 63% had abnormal antigen levels, whereas 75% of women with Stage II disease were marker positive. Three of the 7 stage III patients had elevated levels.

We measured breast tumour marker and CEA serum levels in 39 consecutive patients with metastatic breast cancer. These results are summarized in Table IV. Thirty eight of these patients (97%) showed elevated breast tumour marker levels, but only 46% of the patients had elevated CEA values. CEA monitoring was omitted in subsequent patients.

In 111 patients with metastatic disease, 88% had elevated marker levels. These data are summarized in Figure 3. Patients with metastatic disease had a mean level of 188 U, well above the mean of any other stage. Circulating marker levels in patients with bone lesions are higher than in patients with visceral metastases. Marker levels are highest in patients with both bone and visceral disease. There is, however, significant overlap in marker levels between these groups. The results are summarized in Table V.

The mean age of subjects in our groups was different (Table II) thus we needed to rule out any significant effect of age on marker levels. We first established that there was no significant relation between age of patients and groups ($P=0.24$). Regression analysis which took into account the groups allowed us to establish that there was no significant relation between age and marker levels (age effect: $P=0.27$; group effect: $P<0.0001$). The groups were then compared for mean marker levels using one way analysis of variance. The Student–Neuman–Keuls multiple comparison procedure was applied following one way analysis of variance. This revealed significant differences between the groups ($P<0.0001$). The multiple comparison procedure results are summarized in Table VI. Smoking or non-smoking healthy volunteers and those with benign breast disease did not differ amongst themselves but were different from patients with breast cancer. Amongst those patients with breast cancer there was no difference between those with stage I or II primary disease; both groups were, however, different from patients with metastatic disease.

**Table IV** Sites of disease in patients with elevated CEA

| *Site(s) of metastasis* | *Number of patients* | *CEA positive* | *BTM positive* |
|---|---|---|---|
| Bone | 11 | 5 | 11 |
| Bone and lung | 12 | 5 | 12 |
| Bone and liver | 7 | 5 | 7 |
| Lung | 3 | 1 | 3 |
| Lung and liver | 1 | 0 | 1 |
| Lung, liver and bone | 1 | 0 | 1 |
| Local recurrence | 3 | 1 | 3 |
| Pulmonary lymphangitis | 1 | 1 | 0 |

BTM: Breast tumour marker.

**Table V** Mean marker levels in patients with metastatic breast cancer and the site of metastases at initial diagnosis. Marker values are the mean ± s.d.

| *Sites of metastases* | *% patients* | *Mean antigen value* |
|---|---|---|
| Skin and nodes | 22.9 | $69 \pm 5.7$ U |
| Bones | 36.6 | $214 \pm 225$ U |
| Viscera | 20.7 | $129 \pm 145$ U |
| Viscera and bones | 19.8 | $342 \pm 225$ U |

**Table VI** Student–Neuman–Keuls multiple comparison procedure

| *X* | *Groups* | *HFNS* | *HFS* | *BBD* | *PBI* | *PBII* | *MBC* |
|---|---|---|---|---|---|---|---|
| 2.2767 | HFNS | | | | | | |
| 2.3686 | HFS | | | | | | |
| 2.7768 | BBD | | | | | | |
| 3.4438 | PBI | *** | *** | *** | | | |
| 3.5803 | PBII | *** | *** | *** | | | |
| 4.7291 | MBC | *** | *** | *** | *** | *** | |

X: Mean logarithm of the marker value; *: Indicates groups that differ from each other; HFNS: Healthy females, non smokers; HFS: Healthy females, smokers; BBD: Benign breast disease; PBI: Primary breast cancer, stage I; PBII: Primary breast cancer, stage II; MBC: Metastatic breast cancer.

*Variations in serum marker levels during the course of disease in patients with metastatic breast cancer*

We followed 111 patients with metastatic breast cancer. Patients were divided into three groups: those with disease progression, those with disease regression, and those with stable disease.

We examined the association between changes in marker values and changes in clinical state in patients with metastatic breast cancer using the Kendall tau B test. For this purpose we chose to compare two values of marker level: the value at initial evaluation and the value at the time of clinical determination of change in disease state. (In the case of patients with stable disease the initial serum marker value and the value at the time of the last clinical evaluation were compared. At the close of the trial, two patients with stable disease were noted who had their last serum sample drawn more than 6 weeks prior to completing their clinical reevaluation. These two patients were not reported in Table VII leaving a total of 109 patients for this analysis). This showed an optimal association when a 20% increase or decrease in marker values was the criterion selected (tau = 0.78). These results are summarized in Table VII. Amongst the 62 patients with disease progression, 51 (82%) had increases of >20% in serum marker levels; none had decreases of 20% or more. Thirty patients experienced a regression of their disease; in 22 (73%) there was a larger than 20% decrease in marker levels; no patients showed increases of 20% or more. Four patients with stable disease had a 20% increase in marker level and 2 patients showed a 20% decrease in marker; the other 11 had variations of less than 20%. Overall these data indicate that changes in marker level of 20% are quite specific.

The 6 false positives (the 4 patients with stable disease who show a 20% increase in marker level and the 2 patients with stable disease who show a 20% decrease in marker level) markedly affect the sensitivity of the assay. Five of these false positives had their change in marker value early

**Table VII** Variation in marker levels at time of last documentation of stable disease or of clinical change. Row percentages indicate the % of patients in the category indicated at top of each column

| | *Increase of 20% or more* | *Less than 20% change* | *Decrease of 20% or more* |
|---|---|---|---|
| Patients with progressive disease | 51 pt<br>82% | 11 pt<br>18% | 0 pt<br>0% |
| Patients with stable disease | 4 pt<br>23% | 11 pt<br>65% | 2 pt<br>12% |
| Patients with regression of disease | 0 pt<br>0% | 8 pt<br>27% | 22 pt<br>73% |

on during their follow-up while all their subsequent marker values showed variations of <20%.

The mean initial serum marker levels in the patients who later experienced progression (201 U ± 193.01 s.d.) regression (279 U ± 256 s.d.) or who were stable (57 U ± 48 s.d.) appear quite different. We classified subjects based on the proximity of their initial marker values to the mean marker value of subjects in the three groups. With these criteria only 10, 14 and 16% of patients with progression, regression or stable disease repectively would have been classified correctly. This indicates that initial marker values cannot be used for predicting the clinical course of disease.

We next examined the predictive value of serial assay measurement. These data are summarized in Table VIII. For this purpose we used the criteria of a 20% change in marker value from initial measurement to any one subsequent measurement taken prior to the documented change in disease state or establishment of stable disease. Patients where no serum sample was obtained between entry and time of disease documentation could not be included in this analysis. Twenty five of the 109 patients from Table VII fell into this category.

The two patients with stable disease not included in the Table VII analysis, were, however, eligible for the analysis reported in Table VIII. Eighty percent of patients whose marker level increased by 20% subsequently showed clinical progression of disease. Seventy-nine percent of patients whose marker levels decreased by 20% subsequently showed regression of their disease. One patient with progression experienced a larger than 20% decrease in marker value on one measurement during her follow-up and three patients with regression had one increased value during the early part of their follow-up.

Of the 33 patients with progression where 20% or greater marker variations preceded clinical change (Table VIII), 23 patients (70%) displayed those changes more than 4 weeks prior to progression; in 15 of those 23, marker changes precede progression by more than 8 weeks. Of the 15 patients with regression where significant marker changes preceded clinical change (Table VIII), 6 patients (40%) displayed those decreases more than 4 weeks prior to regression; in 5 of those 6, marker decreases gave lead times of more than 8 weeks.

### Discussion

Serum assays for quantifying tumour markers have been used for monitoring the clinical course of ovarian and gastrointestinal carcinoma (Bast *et al.*, 1983; Del Villano *et al.*, 1983; Klug *et al.*, 1984; Sekine *et al.*, 1985). In breast cancer, the marker that has been most extensively evaluated is CEA. This marker, however, lacks sufficient sensitivity, and variations in serum levels appear to correlate poorly with the course of disease (Chu *et al.*, 1973; Borthwick *et al.*, 1976; Haagensen *et al.*, 1978; De Jong-Bakker *et al.*, 1981). Elevated levels of epithelial membrane antigen (EMA) were

**Table VIII** Variations in marker levels prior to last documentation of stable disease or to clinical change. Row percentages indicate the % of patients in the category shown at the left who had the variation in marker indicated at the top of each column

| | *Increase of 20% or more* | *Less than 20% change* | *Decrease of 20% or more* |
|---|---|---|---|
| Patients with progressive disease | 33 pt 81% | 7 pt 17% | 1 pt 2% |
| Patients with stable disease | 5 pt 26% | 11 pt 58% | 3 pt 16% |
| Patients with regression of disease | 3 pt 11% | 8 pt 31% | 15 pt 58% |

first detected in serum of patients with breast cancer using polyclonal antibodies (Ceriani *et al.*, 1982). More recently monoclonal antibodies reactive with specific components of the EMA complex have been used to detect these antigens in the sera of patients with breast cancer (Thompson *et al.*, 1983; Burchell *et al.*, 1984; Papsidero *et al.*, 1984; Haynes *et al.*, 1985; Hilkens *et al.*, 1986; Major *et al.*, 1987*a*).

We designed a double antibody radioimmunoassay using two monoclonal antibodies which both react with two specific components of the EMA complex. This combination of antibodies was selected for optimal specificity and sensitivity and the assay was designed to quantify marker levels.

The assay reported here is reproducible, linear from 0 to 90 U and stoichiometric beyond the linear range of the standard curve. Decreasing the serum protein content of samples by diluting in protein-free buffers leads to non-linear relations between marker level and assay signal; we determined that 1:4 dilutions of serum were optimal for reducing background and maintaining assay sensitivity (data not shown). Initially, all samples were diluted 1:4 in buffer and further dilutions were done in 1:4 dilutions of a reference serum (LS) to maintain the stoichiometry of the assay. This represents a technical improvement over other assays that have used end point dilution (Haynes *et al.*, 1985) or log-linear standard curves (Hilkens *et al.*, 1986) for quantifying marker levels. Our assay design simplifies quantitative comparison of marker levels in samples obtained serially and allows the comparison of levels between patient groups.

Pregnant and lactating females, in whom one might expect to find higher than normal levels of antigen due to ductal proliferation and milk fat globule secretion, do not show any increased marker levels. Patients with benign breast disease had elevations of marker levels in 13% of cases; these values overlapped with those seen in patients with breast malignancies. Elevated marker levels are observed in 63% of Stage I and in 75% of Stage II patients with breast cancer. This assay is not sufficiently sensitive or specific for screening asymptomatic individuals.

The antigen recognized by our antibodies is detectable by histochemistry in low levels in secretory epithelia other than breast (Major *et al.*, 1987*a*). We measured marker levels in patients with malignancies originating in these tissues and found only small elevations in cases of widely metastatic disease. Use of the marker would appear to be limited to patients with metastatic breast cancer.

There are several possible explanations for failing to detect elevated levels of marker in patients with primary (or metastatic) disease. The antibodies selected react with more than 90% of primary tumours. However, our immunoelectron microscopy studies (Major *et al.*, 1987*b*) show that *in vivo* primarily breast tumours may contain large amounts of antigen in the cytoplasmic compartment and no detectable antigen at the cell surface. Such tumours may not shed antigen into the circulation. In addition, other investigators have documented the presence of EMA components in circulating immune complexes (Salinas *et al.*, 1987); antigen bound in such complexes might not be detectable.

The initial evaluation of our assay shows a good correlation between changes in marker level and changes in the clinical status of patients with metastatic disease (Table VII). Changes smaller than 20% are predictive of stable disease in less than half the cases. More relevant to clinical practice is that, in about half of the cases studied, significant changes in marker occurred before clinical or laboratory evaluation revealed changes in disease state (Table VIII). This may reflect, in part, the ease of performing at each clinic visit a test requiring only a serum sample. Radiographs and nuclear medicine tests although repeated according to standard medical practice guidelines were not done as frequently. It is beyond the scope of this pilot study to compare the sensitivity of standard tests with the serum marker assay. Marker expression is heterogeneous in tumours (Major *et al.*, 1987*a*). A patient with a tumour containing predominantly non marker secreting cells will show a slower rise in marker level than a patient with a tumour containing mostly marker positive cells. This may explain the few cases where long lag-times for rises in marker levels were observed after clinical documentation of a change in disease state. Nevertheless our pilot study would appear to warrant evaluating usefulness of this assay in clinical practice for monitoring patients with metastatic disease.

The secretarial assistance of Mrs E. Jenkins and critical review by Dr G. Price (McGill Cancer Centre) and Drs P. Band, J. Spinelli, C. Lamb (Cancer Control Agency of British Columbia) are gratefully acknowledged. The help and perseverance of the nurses and secretaries of the different clinics collecting samples was essential to the completion of this project. Statistical analysis was performed by TDJ Services for Research Inc., Montreal, Canada.

Supported by funds from the National Cancer Institute of Canada, the Cancer Research Society, Inc., the Fond de la Recherche en Santé du Québec, the Medical Research Council of Canada and the National Cancer Institute of the USA. RLS is a fellow of the Cancer Research Society Inc. PPM is a scholar of the Medical Research Council of Canada.

### References

BAST, JR., R.C., KLUG, T.L., ST. JOHN, E. & 9 others (1983). A radioimmunoassay using a monoclonal antibody to monitor the course of epithelial ovarian cancer. *New Engl. J. Med.*, **309**, 883.

BORTHWICK, N.M., WILSON, D.W. & BELL, P.A. (1976). Carcinoembryonic antigen (CEA) in patients with breast cancer. *Eur. J. Cancer Clin. Oncol.*, **13**, 171.

BURCHELL, J., WANG, D. & TAYLOR-PAPADIMITRIOU, J. (1984). Detection of the tumor-associated antigen recognized by the monoclonal antibodies HMFG-1 and 2 in serum from patients with breast cancer. *Int. J. Cancer*, **34**, 763.

CERIANI, R.L., SASAKI, M., SUSSMAN, M., WARA, W.M. & BLANK, E.W. (1982). Circulating human mammary epithelial antigen in breast cancer. *Proc. Natl Acad. Sci. USA*, **79**, 5420.

CHU, T.M. & NEMOTO, T. (1973). Evaluation of CEA in human mammary carcinoma. *J. Natl Cancer Inst.*, **51**, 1119.

DE JONG-BAKKER, M., HART, A.A.M., PERSIJN, J.-P. & CLETON, F.J. (1981). Prognostic significance of CEA in breast cancer: A statistical study. *Eur. J. Cancer Clin. Oncol.*, **17**, 1307.

DEL VILLANO, B., BRENNAN, S., BROCK, P. & 8 others (1983). Radioimmunometric assay for a monoclonal antibody-defined tumor marker. CA 19.9. *Clin. Chem.*, **29**, 549.

DION, A.S., MAJOR, P.P. & ISHIDA, M. (1987). Characterization of cross-related antigens of human milk fat globule membrane and breast tumor detected by a new monoclonal antibody panel. In *Immunological Approaches to the Diagnosis and Therapy of Breast Cancer*, Ceriani, R.L. (ed) p. 3. Plenum Press, New York.

DRAPER, N.R. & SMITH, H. (1981). *Applied regression analysis*. John Wiley and Sons, New York.

HAAGENSEN, D.E., KISTER, S.J., VANDEVOORDE, J.P. & 4 others (1978). Evaluation of CEA as a plasma monitor for human breast cancer. *Cancer*, **42**, 1512.

HAYNES, D.F., SEKINE, H., OHNO, T., ABE, M., KEEFE, K. & KUFE, D.W. (1985). Use of a murine monoclonal antibody for detection of circulating plasma DF3 antigen levels in breast cancer patients. *J. Clin. Invest.*, **75**, 1397.

HILKENS, J., KROEZEN, V., BONFRER, J.N.G., DE JONG-BAKKER, M. & BRUNING, P.F. (1986). MAM-6 antigen, a new serum marker for breast cancer monitoring. *Cancer Res.*, **46**, 2582.

KENNEDY, J.W. (1984). Protocol EP5-T. User evaluation of precision performance of clinical chemistry devices: Tentative guidelines. 1984. *National Committee for Clinical Laboratory Standards*. Protocol EP5-T.

KLUG, T.L., BAST, JR., R.C., NILOFF, J.N., KNAPP, R.C. & ZURAWSKI, JR., V.R. (1984). Monoclonal antibody immunoradiometric assay for an antigenic determinant (CA 125) associated with human epithelial ovarian carcinomas. *Cancer Res.*, **44**, 1048.

MAJOR, P.P., KOVAC, P.E., LAVALLÉE, M.L. & KOVALIK, E.C. (1987*a*). Monoclonal antibodies to antigens abnormally expressed in breast cancer. *J. Histochem. Cytochem.*, **35**, 139.

MAJOR, P., LAVALLÉE, M., MINASSIAN, H., KOVAC, P. & WANG, N.-S. (1987*b*). Ultrastructural localization of a breast tumor-associated antigen. *J. Histochem. Cytochem.*, **35**, 375.

PAPSIDERO, L.D., NEMOTO, T., CROGHAN, G.A. & CHU, T.M. (1984). Expression of ductal carcinoma antigen in breast cancer sera as defined using monoclonal antibody F36-22. *Cancer Res.*, **44**, 4653.

SALINAS, F.A., WEE, K.H. & CERIANI, R.L. (1987). Significance of breast carcinoma-associated antigens as a monitor of tumor burden: Characterization by monoclonal antibodies. *Cancer Res.*, **47**, 907.

SEKINE, H., HAYES, D.F., OHNO, T. & 5 others (1985). Circulating DF3 and CA125 antigen levels in serum from patients with epithelial ovarian carcinoma. *J. Clin. Oncol.*, **3**, 1355.

SOKAL, P.R. & ROHLF, F.J. (1981). *Biometry*. W.H. Freeman and Company, San Francisco.

SWENERTON, K.D., SEWA, S.S., SMITH, T. & 5 others (1979). Prognostic factors in metastatic breast cancer treated with combination chemotherapy. *Cancer Res.*, **39**, 1552.

THOMPSON, C.H., JONES, S.C., WHITEHEAD, R.M. & MCKENZIE, I.F.C. (1983). A human breast tissue-associated antigen detected by a monoclonal antibody. *J. Natl Cancer Inst.*, **70**, 409.